# Evidence of Gene−Environment Interaction for Two Genes on Chromosome 4 and Environmental Tobacco Smoke in Controlling the Risk of Nonsyndromic Cleft Palate

**DOI:** 10.1371/journal.pone.0088088

**Published:** 2014-02-06

**Authors:** Tao Wu, Holger Schwender, Ingo Ruczinski, Jeffrey C. Murray, Mary L. Marazita, Ronald G. Munger, Jacqueline B. Hetmanski, Margaret M. Parker, Ping Wang, Tanda Murray, Margaret Taub, Shuai Li, Richard J. Redett, M. Daniele Fallin, Kung Yee Liang, Yah Huei Wu-Chou, Samuel S. Chong, Vincent Yeow, Xiaoqian Ye, Hong Wang, Shangzhi Huang, Ethylin W. Jabs, Bing Shi, Allen J. Wilcox, Sun Ha Jee, Alan F. Scott, Terri H. Beaty

**Affiliations:** 1 Peking University Health Science Center, Beijing, China; 2 Johns Hopkins University, School of Public Health, Baltimore, Maryland, United States of America; 3 Mathematical Institute, Heinrich Heine University Duesseldorf, Duesseldorf, Germany; 4 University of Iowa, Children’s Hospital, Iowa City, Iowa, United States of America; 5 Center for Craniofacial and Dental Genetics, School of Dental Medicine, University of Pittsburgh, Pittsburgh, Pennsylvania, United States of America; 6 Utah State University, Logan, Utah, United States of America; 7 Johns Hopkins University, School of Medicine, Baltimore, Maryland, United States of America; 8 National Yang-Ming University, Taipei, Taiwan; 9 Chang Gung Memorial Hospital, Taoyuan, Taiwan; 10 National University of Singapore, Singapore, Singapore; 11 KK Women’s & Children’s Hospital, Singapore, Singapore; 12 Wuhan University, School of Stomatology, Wuhan, China; 13 Mount Sinai Medical Center, New York, New York, United States of America; 14 Peking Union Medical College, Beijing, China; 15 State Key Laboratory of Oral Disease, West China College of Stomatology, Sichuan University, Chengdu, China; 16 NIEHS/NIH, Epidemiology Branch, Durham, North Carolina, United States of America; 17 Yonsei University, School of Public Health, Seoul, Korea; Wake Forest University Health Sciences, United States of America

## Abstract

Nonsyndromic cleft palate (CP) is one of the most common human birth defects and both genetic and environmental risk factors contribute to its etiology. We conducted a genome-wide association study (GWAS) using 550 CP case-parent trios ascertained in an international consortium. Stratified analysis among trios with different ancestries was performed to test for GxE interactions with common maternal exposures using conditional logistic regression models. While no single nucleotide polymorphism (SNP) achieved genome-wide significance when considered alone, markers in *SLC2A9* and the neighboring *WDR1* on chromosome 4p16.1 gave suggestive evidence of gene-environment interaction with environmental tobacco smoke (ETS) among 259 Asian trios when the models included a term for GxE interaction. Multiple SNPs in these two genes were associated with increased risk of nonsyndromic CP if the mother was exposed to ETS during the peri-conceptual period (3 months prior to conception through the first trimester). When maternal ETS was considered, fifteen of 135 SNPs mapping to *SLC2A9* and 9 of 59 SNPs in *WDR1* gave *P* values approaching genome-wide significance (10^−6^<*P*<10^−4^) in a test for GxETS interaction. SNPs rs3733585 and rs12508991 in *SLC2A9* yielded *P* = 2.26×10^−7^ in a test for GxETS interaction. SNPs rs6820756 and rs7699512 in *WDR1* also yielded *P* = 1.79×10^−7^ and *P* = 1.98×10^−7^ in a 1 *df* test for GxE interaction. Although further replication studies are critical to confirming these findings, these results illustrate how genetic associations for nonsyndromic CP can be missed if potential GxE interaction is not taken into account, and this study suggest *SLC2A9* and *WDR1* should be considered as candidate genes for CP.

## Introduction

Nonsyndromic cleft palate (CP) is a common birth defects and has a complex and heterogeneous etiology, involving both genetic and environmental risk factors [1]. The prevalence of CP is about 1/2500 live births, much lower than the 1/1000 live births prevalence for nonsyndromic cleft lip with or without cleft palate (CL/P). About half of all CP cases have another congenital anomaly or represent a recognized malformation syndrome, with the remaining half representing isolated nonsyndromic CP cases [2].

Genetic risk factors play an important role in the etiology of CP. A recent twin study in Denmark showed heritability of CP is as high as 90%, and the proband-wise concordance rate for CP among monozygotic twins was much higher compared to dizygotic twins: 33% vs. 7% [3]. Both family studies and population based studies have identified multiple candidate genes associated with increased risk of CP, including *FOXE1*, *ALX3*, *MKX*, *PDGFC*, and *SUMO1* [4]–[6]. However, evidence of association between reported candidate genes and CP remains inconsistent. Compared to the candidate gene approach, genome wide association studies (GWAS) have the advantage of providing better coverage of the human genome and are unbiased from a genetic perspective. Although several GWAS have identified strong signals at several chromosomal regions in multiple populations for CL/P [7]–[11], the variants controlling risk of CP have proven more difficult to find.

A few studies of CP have investigated potential GxE interaction for candidate genes and maternal exposure to cigarette smoking [12]–[14]. Environmental tobacco smoke (ETS) has also been reported to interact with certain SNPs to influence the risk of nonsyndromic CL/P and CP [15]–[20]. However, the evidence of GxE interaction has been rather inconclusive [21]. Possible reasons for the difficulty in documenting potential GxE interactions include: limited power due to modest sample size, different study designs and lack of available replication data. Integrating GxE interaction analysis into GWAS design is a powerful strategy for identifying more genetic factors influencing risk of complex disease, which could be overlooked when such interaction is ignored. A recent GWAS using the case-parent trio design found markers in several genes (*MLLT3*, *SMC2*, *TBK1*, *ZNF236*, and *BAALC*) showed statistically significant interaction with common maternal exposures, although no single SNP achieved genome-wide significance when such GxE interaction was ignored [22]. Beaty et al. (2011) combined CP trios from 12 different recruitment sites in their analysis, which involved considerably different rates of exposure to certain maternal exposures [22]. While the case-parent trio design has the advantage of being robust to confounding due to population stratification (compared to case-control designs), therefore allowing multi-site studies to amass large sample sizes, this advantage may not hold when considering GxE interaction especially if the exposure rates vary across sites.

In this study, we performed stratified analysis of the CP case-parent trios from the International Cleft Consortium [22] among trios with different ancestries to test for GxE interactions with common maternal exposures, including maternal cigarette smoking, alcohol consumption, ETS and multivitamin supplementation. Here we classified the trios used by Beaty et al. (2011) into groups of Asian and European ancestry and explored the potential GxE interactions.

## Subjects and Methods

### Case-parent Trios

Research protocols were reviewed and approved by institutional review boards (IRB) at each institution, including IRBs at The Johns Hopkins School of Public Health, University of Iowa, University of Pittsburgh, Utah State University, and all foreign collaborators. The review process for the consortium was approved by Johns Hopkins’ IRB. Written informed consent was obtained from parents. Case-parent trios were drawn from an international consortium which conducted a GWAS using a case-parent trio design to search for genes controlling risk of nonsyndomic, isolated oral clefts [10]. Most cases were ascertained through surgical treatment centers at a surgical or post-surgical visit. Racial/ethnic background of participants was originally based on self-report and most of the 550 CP trios were of European or Asian ancestry, but this was confirmed by genotyping. Table 1 lists charateristics of the CP probands noting gender and recruitment site, stratified by European or Asian ancestry. To minimize potential misclassification of nonsyndromic CP, all probands were examined for other congenital anomalies or major developmental delays by either a clinical geneticist or experienced health care provider to rule out syndromic forms of oral clefts. As expected, there were slightly more female CP cases (56.1%) compared to males. None of the parents of these CP cases were themselves affected.

**Table 1:** Gender of isolated, nonsyndromic cleft palate (CP) cases in the International Cleft Consortium by recruitment site.

Asian ancestry			
Site	Males	Females	Total
Singapore	20	30	50
Taiwan	29	50	79
Shangdong Prov,China	16	22	38
Hubei Prov., China	19	26	45
Sichuan Prov., China	18	22	40
Other*	4	3	7
Subtotal	106 (40.9%)	153 (59.1%)	259
**European ancestry**			
**Site**	**Males**	**Females**	**Total**
Denmark	8	5	13
Norway	52	58	110
Iowa	18	22	40
Maryland	14	21	35
Pittsburg	6	8	14
Utah	29	27	56
Singapore	1	3	4
Subtotal	128(47.1%)	144(52.9%)	272
Other ancestries**			
	10(52.0%)	9(48.0%)	19
**Total**	**244(43.9%)**	**306(56.1%)**	**550**

*other sites include Maryland, Utah, and Korea.

**other ancestries include African American, Hispanic, Malay and others.

### Genotyping

The Center for Inherited Disease Research (CIDR) genotyped DNA samples using Illumina’s 610 Quad platform and 99.1% passed CIDR quality control (QC) [10]. Genotypes on 589,945 SNPs (99.56% of those attempted) were released and then underwent further QC analysis to set up 4 types of QC flags for each SNP: 1) unacceptably high rates (>5%) of missing genotype calls, 2) low minor allele frequency (MAF<0.01), 3) unacceptably high rates of Mendelian errors (>5%) between parents and child, and 4) significant deviation (p<10^−5^) from Hardy Weinberg equilibrium (HWE) among parents within recruitment site or across European and Asian populations separately. This QC process flagged 14.6% of all SNPs (mostly for low MAF), leaving ∼498 K SNPs available for analysis.

### Exposure Assessment

Maternal exposure information, including cigarette smoking, ETS, multivitamin supplementation, and alcohol consumption was collected through direct interview of mothers. Only Asian sites collected complete information on ETS. Environmental exposures were defined as being exposed from three months prior to pregnancy through the first trimester. The question measuring ETS status during certain periods asked “did someone smoke in your home, workplace or any other place near you?”. Maternal exposures were assessed as simple yes/no responses. See Table II in Beaty et al. (2011) for details of the exposure rates for all CP trios. The proportion of infants exposed to maternal cigarette smoking and alcohol consumption was very low among Asian mothers (around 4%), so only maternal ETS and multivitamin supplementation could be analyzed in this group. The proportion of exposure to ETS and multivitamin supplementation among Asians were 40.3% and 20.2%, respectively.

### Statistical Analysis

MAFs were computed using parents only. Pairwise linkage disequilibrium (LD) was measured as r^2^ for all SNPs using the Haploview program, and was used to identify linkage disequilibrium (LD) blocks [23]. In this study, we used a closed form genotypic transmission disequilibrium test (gTDT) developed by Schwender et al. (2012) to test for genetic association of each SNP [24]. To perform this gTDT, a “pseudo-control” dataset was created based on the observed genotype of the case and all alternative possible genotypes given the parental mating type. The gTDT has a number of advantages compared to allelic TDT [25]. While assuming different models of inheritance, the gTDT can be used to estimate the relative risks (RRs) of each genotype and a term for GxE interaction can also be incorporated. Schwender et al. (2012) developed a method with a closed form solution providing parameter estimates for genome-wide markers efficiently [24] and is implemented in the R package Trio (v 1.5.0).

All autosomal markers were examined using the conditional logistic regression model assuming an additive model of inheritance. The log-odds of being the observed case in the i-th trio is modeled as: logit[P(case_i_)] = β_G_(G_i_)+ β_GxE_(G_i_xE_i_), where G = 0, 1, or 2 stands for the number of risk alleles in the case:“pseudo-control” set (representing a 1∶3 matching), andwhere E = 0 or 1 reflects unexposed or exposed mothers, respectively. A 2 degree of freedom (*df*) likelihood ratio test (LRT) for joint effects of G and GxE interaction was first performed, followed by a 1 *df* LRT for GxE interaction alone. The 2 *df* test examines the inherited effect of the SNP after taking into account effects of GxE interaction, while the 1 *df* test focuses exclusively on GxE interaction. We used RR(CP|G no E) = exp(β_G_) to represent the estimated RRs of being a case with one copy of the risk allele in the absence of maternal environment exposures, while RR(CP|G and E) = exp(β_G_+β_GxE_) reflects the RR of being a case carrying one copy of the risk allele in the presence of maternal exposure.

## Results

A conventional search for marginal gene (G) effects in the total sample of 550 CP trios, as well as in the stratified analysis of trios of Asian and European ancestry, showed no markers achieved significance at a genome-wide level (*P*≤10^−7^, data not shown).

**Figure 1:**
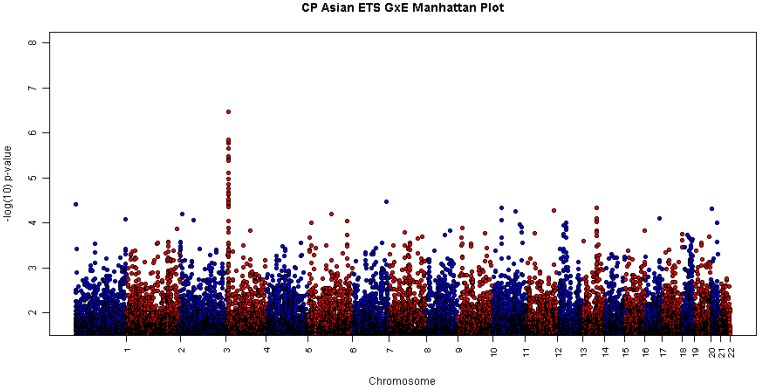
CP Asian ETS G×E Manhattan Plot. Manhattan plot with *P* values from likelihood ratio tests with 1 degree of freedom testing for GxETS interaction among 259 Asian CP trios (492,698 SNPs were left in Asian trios after quality control).

A genome-wide screen for GxE interaction was carried out using Trio (1.5.0), where conditional logistic regression models were used to estimate effects of GxE interaction alone (LRT with 1 *df* ), as well as the combined effects of gene (G) and gene-environment (GxE) interaction (LRT with 2 *df*). This screening process yielded no significant signals among European trios (see Figure S1 in File S1 for GxE interaction results on maternal smoking, alcohol consumption and multivitamin supplementation among European trios), but revealed several markers with suggestive evidence of GxE interaction (10^−6^<*P*<10^−4^) among 259 Asian trios clustered on chromosome 4p16, especially in the 1 *df* test for GxETS interaction. Figure 1 presents a conventional Manhattan plot for all autosomal SNPs where –log_10_(*P*) from the 1 *df* LRT for GxETS interaction was plotted (See Figure S2 in File S1 for a Q-Q plot of GxETS interaction among Asian trios). Therefore, we mainly present results for GxETS interaction among Asian trios here.

**Figure 2:**
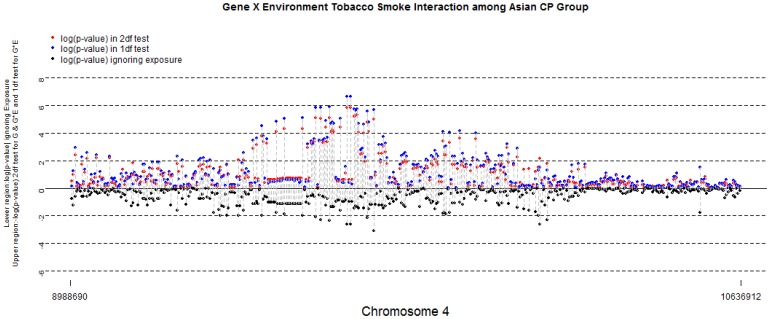
Gene × Environmental Tobacco Smoke Interaction among Asian CP Group. Double Manhattan plots for SNP effects ignoring maternal exposures (black dots in the lower half) and considering G and GxE interaction for environmental tobacco smoke on selected region on chromosome 4p among 259 Asian trios. Blue dots represent -log_10_(P) from the 1 df test of GxE interaction alone; red dots represent -log_10_ (P) from the 2 df test of G and GxE interaction. Dashed lines connect SNP showing this level of significance in one test considering GxE interaction with their corresponding P-value when interaction was ignored.

To further investigate this evidence, Figure 2 presents a “double Manhattan plot” to summarize joint evidence for G and GxETS interaction effects on chromosome 4p (over the region 8988690 kb∼10636912 kb). Table S1 in File S1 showed the physical location and MAFs of SNPs in this region (19 SNPs with MAF <0.01 were dropped in this region). The bottom half of this plot shows the log_10_(*P*) for the conventional family-based test of SNP effects *ignoring exposure* (where more significant results fall farther below the mid-line). In the top half of Figure 2, –log_10_(*P*) are shown for each autosomal SNP from both the 2 *df* test of G and GxE interaction together (red dots) and the 1 *df* test for GxE interaction alone (blue dots). Dashed lines connect *P*-values from the marginal test ignoring exposures (below the mid-line) to those models considering GxE interaction (above the mid-line). As seen in Figure 2, more than 20 markers gave *P* values approaching genome-wide significance level in tests for GxETS interaction, including 15 SNPs in *SLC2A9* and 9 in *WDR1* on chr. 4p16.1 (Figure S3 in File S1 shows LD plots for these two genes).

Although none of 135 SNPs mapping to *SLC2A9* approached genome-wide significance level when maternal exposure to ETS was ignored (lower half of Figure 2), a cluster of 61 SNPs identified a region spanning 125 kb yielded *P* values approaching genome-wide significance levels when interaction with maternal ETS was considered. In this region, fifteen SNPs showed suggestive evidence of GxETS interaction in the 1 *df* test (Table 2). SNPs rs3733585 and rs12508991 suggested GxETS interaction in the 1 *df* test (*P = *2.26×10^−7^).

**Table 2:** Estimated RR(case|G no E) and RR(case|G and E) from conditional logistic regression using cases and 3 pseudo-controls in 259 Asian CP case-parent trios for 15 SNPs in *SLC2A9* considering GxE interaction between each SNP and maternal exposure to environmental tobacco smoke.

SNP	Physicallocation	TA (freq)	RR(case|G no E)	RR(case|G and E)	LRT 2 *df P* values	LRT 1 *df P* values
rs4447863	9548067	C(0.593)	0.64(0.46,0.90)	2.07(1.32,3.25)	1.61×10^−4^	2.94×10^−5^
rs998676	9557662	G(0.587)	0.64(0.46,0.90)	2.07(1.32,3.25)	7.51×10^−5^	1.36×10^−5^
rs6849717	9567817	C(0.590)	0.66(0.47,0.91)	2.26(1.44,3.55)	5.07×10^−5^	9.01×10^−6^
rs11723970	9589560	T (0.588)	0.64(0.46,0.90)	2.21(1.42,3.46)	4.67×10^−5^	8.14×10^−6^
rs17187075	9599426	G(0.596)	0.64(0.46,0.89)	2.48(1.56,3.95)	8.22×10^−6^	1.38×10^−6^
rs12499857	9604474	G(0.600)	0.62(0.44,0.86)	2.46(1.53,3.95)	8.57×10^−6^	1.36×10^−6^
rs10939650	9607538	T(0.500)	0.71(0.50,1.01)	2.46(1.56,3.88)	3.91×10^−5^	1.22×10^−5^
rs4622999	9612493	C(0.600)	0.61(0.44,0.86)	2.46(1.53,3.95)	8.08×10^−6^	1.28×10^−6^
rs7657096	9613098	A(0.528)	0.70(0.49,0.99)	2.33(1.49,3.66)	7.74×10^−5^	2.03×10^−5^
rs10022499	9615635	C(0.492)	0.71(0.50,1.00)	2.37(1.51,3.72)	5.87×10^−5^	1.60×10^−5^
rs10016075	9615761	G(0.492)	0.71(0.50,1.00)	2.33(1.49,3.66)	8.24×10^−5^	2.15×10^−5^
rs2240723	9630249	A(0.465)	0.67(0.47,0.95)	2.32(1.49,3.62)	3.93×10^−5^	8.81×10^−6^
rs3733585	9645437	C(0.412)	0.60(0.43,0.83)	2.58(1.61,4.14)	1.52×10^−6^	2.26×10^−7^
rs12508991	9650202	C(0.588)	0.60(0.43,0.83)	2.58(1.61,4.14)	1.52×10^−6^	2.26×10^−7^
rs733175	9659239	C(0.488)	0.69(0.49,0.98)	2.71(1.70,4.33)	6.12×10^−6^	1.89×10^−6^

TA: target allele and its frequency among parents of Asian ancestry.

**Figure 3:**
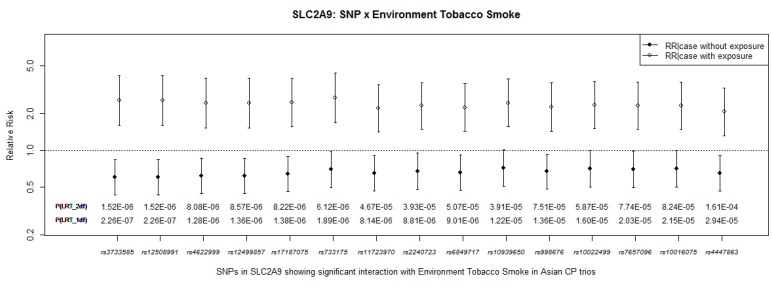
SLC2A9: SNP × Environmental Tobacco Smoke. Estimated RR(CP|G no E) and RR(CP|G and E) from conditional logistic regression model considering SNP effects and their interaction with maternal exposure to ETS on 259 CP case-parent trios of Asian ancestry for fifteen SNPs in *SLC2A9*. P-values from the 2 *df* and 1 *df* LRT for GxE interaction are shown along the X axis.

Regression coefficients from the conditional logistic regression model provide an estimate of exposure specific RRs under this additive model. When both G and GxE terms were included in the conditional logistic regression model, RRs were also calculated for both exposed and unexposed heterozygous carriers of the apparent risk allele. Figure 3 shows estimated RR(CP|G no E) and RR(CP|G and E) for 15 SNPs in *SLC2A9* along with *P* values from the LRT for both the 2 *df* and 1 *df* test. Here, the apparent “risk allele” became the target allele (which was the minor allele for rs10022499, rs10016075, rs2240723, rs3733585, rs733175, but the major allele for rs4447863, rs998676, rs6849717, rs11723970, rs17187075, rs12499857, rs10939650, rs4622999, rs7657096, rs12508991–see Table 2). Estimated RR(CP|G and E) and their 95%*CI* for a heterozygous child whose mother was exposed to ETS were distinctly higher (open circles) compared to a similar heterozygous child of unexposed mothers (solid circles). For the two most significant SNPs (rs3733585 and rs12508991) being a heterozygous child of an exposed mother was associated with a 2.58-fold increase in risk (RR = 2.58; 95% CI: 1.61–4.14), but not among children of unexposed mothers (RR = 0.60; 95% CI:0.43–0.83). The 1 *df* LRT for GxETS interaction in this conditional logistic regression model approached genome-wide significance (*P* = 2.26×10^−7^).

**Figure 4:**
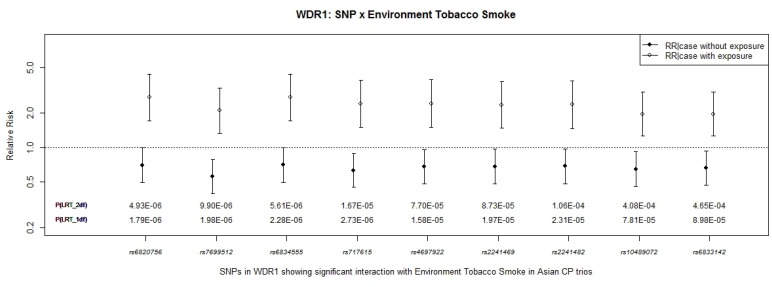
WDR1: SNP × Environmental Tobacco Smoke. Estimated RR(CP|G no E) and RR(CP|G and E) from conditional logistic regression model considering SNP effects and their interaction with maternal exposure to ETS on 259 CP case-parent trios of Asian ancestry for nine SNPs in *WDR1*. P-values from the 2 *df* and 1 *df* LRT for GxE interaction are shown along the X axis.

*WDR1* on chr. 4p16.1 is located next to *SLC2A9* and encompasses 59 SNPs. Like *SLC2A9*, none of these SNPs achieved genome-wide significance levels alone, however, a block of 9 SNPs (spanning 213 kb) showed suggestive GxETS interaction in the 1 *df* test for GxE interaction (Table 3). SNPs rs6820756 and rs7699512 yielded *P* = 1.79×10^−6^ and *P* = 1.98×10^−6^ in the 1 *df* test for GxE interaction, and two adjacent SNPs also approached genome-wide significance (rs6834555, *P* = 2.28×10^−6^; rs6834555, *P* = 2.73×10^−6^). Figure 4 shows estimated RR(CP|G no E) and RR(CP|G and E), plus their 95%CI, for these 9 SNPs under an additive model. The risk of having nonsyndromic CP was 1.97–2.75 times higher when the fetus carried the risk allele and the mother was exposed to ETS compared to carriers whose mothers were not exposed.

**Table 3:** Estimated RR(case|G no E) and RR(case|G and E) from conditional logistic regression using cases and 3 pseudo-controls in 259 CP case-parent trios for 9 SNPs in *WDR1* considering maternal exposure to environmental tobacco smoke.

SNP	Physical location	TA (freq)	RR(case|G no E)	RR(case|G and E)	LRT 2 df p-value	LRT 1 df p-value
rs6834555	9671424	G(0.482)	0.71(0.50,1.00)	2.75(1.72,4.39)	5.61×10^−6^	2.28×10^−6^
rs6820756	9671947	A(0.482)	0.70(0.50,0.99)	2.75(1.72,4.39)	4.93×10^−6^	1.79×10^−6^
rs2241469	9689560	A(0.652)	0.68(0.48,0.97)	2.36(1.48,3.77)	8.73×10^−5^	1.97×10^−5^
rs2241482	9708912	G(0.654)	0.69(0.49,0.98)	2.37(1.47,3.83)	1.06×10^−4^	2.31×10^−5^
rs717615	9713768	C(0.527)	0.63(0.45,0.88)	2.42(1.50,3.89)	1.67×10^−5^	2.73×10^−6^
rs4697922	9719703	C(0.657)	0.68(0.48,0.97)	2.43(1.50,3.96)	7.70×10^−5^	1.58×10^−5^
rs7699512	9734906	T(0.519)	0.56(0.40,0.79)	2.11(1.34,3.30)	9.90×10^−6^	1.98×10^−6^
rs10489072	9882342	G(0.499)	0.65(0.46,0.92)	1.97(1.27,3.05)	4.08×10^−4^	7.81×10^−5^
rs6833142	9885080	G(0.494)	0.66(0.47,0.93)	1.97(1.27,3.05)	4.65×10^−4^	8.98×10^−5^

TA: target allele and its frequency among parents of Asian ancestry.

Examining the imputed genotypes generated by the GENEVA Coordinating Center [26] using 1000 Genomes reference populations after pre-phasing haplotypes using IMPUTE2 [27] yielded additional evidence of GxETS interaction. Analysis of imputed SNPs in the region of these two genes yielded genome-wide significance for several markers (see Figure S4 in File S1).

Similar analysis for potential GxE interaction with maternal multivitamin supplementation in these same Asian CP trios showed no significant GxE interaction. Because the exposure rate for maternal multivitamin supplementation was lower in this sample of Asian CP trios (∼20%), however, this sample had less statistical power to detect GxE interaction unless the causal allele were highly polymorphic (MAF>0.15) and the true interaction effects were at least as large as those seen in test of GxETS interaction (RR_GE_>2.5).

## Discussion

While the initial GWAS of 550 CP case-parent trios stratified by European and Asian ancestry did not yield any markers achieving genome-wide significance (i.e. when GxE interaction was ignored), multiple markers in two adjacent genes on chr. 4p16.1 (*SLC2A9* and *WDR1)* showed *P*-values approaching genome-wide significance when GxETS interaction was incorporated into the analysis of Asian trios. Our results suggested *SLC2A9* and/or *WDR1* located at position 9 Mb on chromosome 4p16.1 may influence risk of nonsyndromic CP through interaction with maternal exposure to ETS, though independent replication studies are still needed to confirm these findings. Our study did not yield any compelling evidence of GxE interactions approaching genome-wide significance among trios of European ancestry.

Identifying GxE interaction will lead to better understanding of the etiology of common birth defects and potential biological mechanisms, as well as create opportunities for designing effective prevention strategies. Several studies have shown maternal smoking is not only an independent risk factor for CP [28], [29], but may interact with genetic variants to influence risk [12]–[14]. GxSmoking interaction has been suggested for markers in the chr. 4p16 region. A previous case-control study and case-parent trio studies showed evidence of GxSmoking for markers near *MSX1* on chr. 4p16 among CP trios or combined CL/P and CP trios [30], [31]. This 4p16 region has been suggested to be associated with increased risk of nonsyndromic oral clefts, including CL/P and CP in a previous analysis [32]. Ingersoll et al. (2010) used 381 case-parent trios from four populations including Asian samples from Singapore, Korea and Taiwan [32]. Their analysis focused on the 2 Mb region around *MSX1* and showed SNP effects in *STK32B*, the *EVC–EVC2–CRMP1* region, and the *STX18–MSX1* region were significantly associated with risk to CP, especially among Asian trios. A Dutch study showed smoking by both parents may interact with SNPs in *MSX1* to increase the risk of nonsyndromic oral clefts [16]. *SLC2A9* and *WDR1*, the most significant genes seen here, are located about 3 Mb downstream of *MSX1*. ETS has been shown to interact with candidate genes to influence risk of nonsyndromic oral clefts in different populations. Previous studies have shown ETS may interact with *IRF6*, *RUNX2*, and *BMP4* among Chinese CL/P case-parent trios [17], [19], [33]. Another French study yielded suggestive evidence for interaction between *CYP1A1* and ETS among nonsyndromic oral cleft trios [15]. Li et al. (2011) also found maternal ETS interacted with one SNP in *microRNA-140* gene to increase the risk of nonsyndromic CP using case-control design in a Chinese population [18].

The *WDR1* gene (WD repeat domain 1, also called actin-interacting protein 1) is downstream from *SLC2A9* and it is highly conserved in eukaryotes and promotes cofilin-mediated actin filament disassembly [34]. Kato et al. (2008) noted *WDR1* has an important role in unidirectional cell migration by promoting cofilin activity. Protein aggregates of actin and cofilin in the brains of twins with dystonia and CL/P were described by Gearing, et al. (2010) [35]. While neither of these studies is proof of any link between *WDR1* and nonsyndromic oral clefts, they suggest a possible biological mechanism involving disruption of cell migration during development of the palate. The *SLC2A9* gene (solute carrier family 2, member 9) is located on chromosome 4p16.1, and encodes a member of the *SLC2A* facilitative glucose transporter gene family, which is critical for maintaining glucose homeostasis. Multiple association studies across several populations showed consistent findings that this gene is associated with uric acid concentration and risk of gout [36]–[43], with a higher effect size among females compared to males. In our study, markers in these genes were in high LD. Therefore, the significant findings of GxETS interaction in *SLC2A9* may reflect its close physical proximity to *WDR1*. We also performed the GxETS analysis using imputed genotype data in this chromosomal region among Asian trios, and the imputed genotypes yielded greater significance (including several achieving genome-wide significance) in the region (Figure S4 in File S1). In addition, we tested for parent-of-origin effects among exposed and unexposed trios using the parent-of-origin likelihood ratio test [44], but found no significant signals. Our study suggested genes in this region may play a role in the etiology of CP not only through gene effects but also may through potential GxE interactions, at least among Asian populations. Although it is unclear how either of these genes affects cleft development, our results suggest these two genes (especially *WDR1*) should be considered as candidate genes for nonsyndromic CP.

We acknowledge the suggestive GxETS interactions on chr. 4p16.1 region seen in the present study require further confirmation in independent samples. However, adequate sample size will be a challenge. Our results argue maternal ETS appears to increase risk of nonsyndromic CP in Asian cases carrying certain genotypes in *SLC2A9* and *WDR1*. Exposure rate of ETS among Asian mothers in this sample was as high as 40%, reflecting the high prevalence of smoking among Asian males (about 60%) [45]. If this observation can be confirmed, such a GxE interaction creates opportunities for an effective intervention to reduce the risk. Our suggestive evidence of interaction between ETS and two genes on Chromosome 4 would be strengthened if we could test for GxSmoking interaction in this same population. However, such analysis would be severely underpowered due to low rates of personal smoking among Asian women. Further analyses will be required to understand how maternal exposure to ETS could interact with genes to affect fetal development. In addition, the analysis testing for the interaction between maternal genes and environmental exposures could also be informative. While the case-parent trio design is robust to population stratification [46], [47], and stratification into Asian/European ancestries minimizes potential confounding due to differences in exposure rates, this study illustrates the importance of considering possible GxE interaction in the etiology of CP. Still statistical interaction does not guarantee biological interaction, and the functional gene may be located some distance from the statistical signals for GxE interaction seen here.

## Supporting Information

File S1 **Supporting information.** Figure S1.1: Manhattan plot with *P* values from likelihood ratio tests with 1 degree of freedom testing for GxSmoking interaction among 272 European CP trios. Figure S1.2 Manhattan plot with *P* values from likelihood ratio tests with 1 degree of freedom testing for GxAlcohol consumption interaction among 272 European CP trios. Figure S1.3 Manhattan plot with *P* values from likelihood ratio tests with 1 degree of freedom testing for GxMultivitamin supplementation consumption interaction among 272 European CP trios. Figure S2: Q-Q plot with *P* values from likelihood ratio tests with 1 degree of freedom testing for GxETS interaction among 259 Asian CP trios (492,698 SNPs were left in Asian trios after quality control). The gray shaded region indicates 95% confidence band for order statistics. The numbers on the top axis indicate the respective locations for (ordered) expected –log10 p-values. (e.g., the number 1 (10) indicates the expected value, on the –log10 scale, for the minimum (i.e. the tenth smallest) p-value). Figure S3.1 LD plots for *SLC2A9* among 259 Asian CP trios. Black squares represent r^2^ = 1; gray squares represent 0<r^2^<1; white squares represent r^2^ = 0. Figure S3.2 LD plots for *WDR1* among 259 Asian CP trios. Black squares represent r^2^ = 1; gray squares represent 0<r^2^<1; white squares represent r^2^ = 0. Figure S4: *P* values from likelihood ratio test with 1 degree of freedom testing for GxETS interaction after including the imputed SNPs among Asian CP trios. Circles represent imputed genotypes using 1000 Genomes as a reference population and squares represent observed SNPs. Table S1.
(DOCX)
