# Supplementary material for: Evidence of Gene−Environment Interaction for Two Genes on Chromosome 4 and Environmental Tobacco Smoke in Controlling the Risk of Nonsyndromic Cleft Palate
Source: PLoS One. 2014 Feb 6;9(2):e88088. doi: 10.1371/journal.pone.0088088 (PMC3916361; doi:10.1371/journal.pone.0088088)
Supplement: File S1 — Supporting information. Figure S1.1: Manhattan plot with P values from likelihood ratio tests with 1 degree of freedom testing for GxSmoking interaction among 272 European CP trios. Figure S1.2 Manhattan plot with P values from likelihood ratio tests with 1 degree of freedom testing for GxAlcohol consumption interaction among 272 European CP trios. Figure S1.3 Manhattan plot with P values from likelihood ratio tests with 1 degree of freedom testing for GxMultivitamin supplementation consumption interaction among 272 European CP trios. Figure S2: Q-Q plot with P values from likelihood ratio tests with 1 degree of freedom testing for GxETS interaction among 259 Asian CP trios (492,698 SNPs were left in Asian trios after quality control). The gray shaded region indicates 95% confidence band for order statistics. The numbers on the top axis indicate the respective locations for (ordered) expected –log10 p-values. (e.g., the number 1 (10) indicates the expected value, on the –log10 scale, for the minimum (i.e. the tenth smallest) p-value). Figure S3.1 LD plots for SLC2A9 among 259 Asian CP trios. Black squares represent r2 = 1; gray squares represent 0<r2<1; white squares represent r2 = 0. Figure S3.2 LD plots for WDR1 among 259 Asian CP trios. Black squares represent r2 = 1; gray squares represent 0<r2<1; white squares represent r2 = 0. Figure S4: P values from likelihood ratio test with 1 degree of freedom testing for GxETS interaction after including the imputed SNPs among Asian CP trios. Circles represent imputed genotypes using 1000 Genomes as a reference population and squares represent observed SNPs. Table S1. (DOCX) [file pone.0088088.s001.docx]

Figure S1.1: Manhattan plot with *P* values from likelihood ratio tests with 1 degree of freedom testing for GxSmoking interaction among

272 European CP trios.

Figure S1.2 Manhattan plot with *P* values from likelihood ratio tests with 1 degree of freedom testing for GxAlcohol consumption interaction among

272 European CP trios.

Figure S1.3 Manhattan plot with *P* values from likelihood ratio tests with 1 degree of freedom testing for GxMultivitamin supplementation consumption interaction among 272 European CP trios.


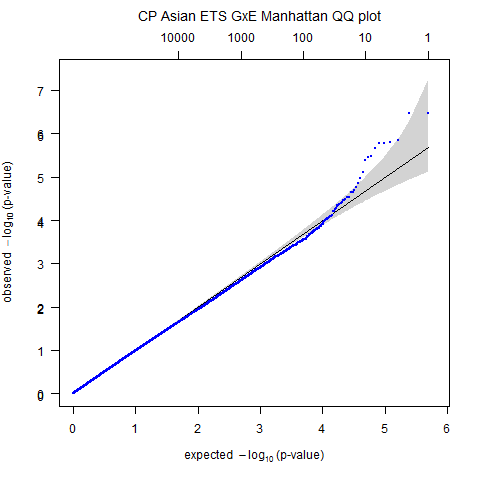


Figure S2: Q-Q plot with *P* values from likelihood ratio tests with 1 degree of freedom testing for GxETS interaction among 259 Asian CP trios (492,698 SNPs were left in Asian trios after quality control). The gray shaded region indicates 95% confidence band for order statistics. The numbers on the top axis indicate the respective locations for (ordered) expected –log10 p-values. (e.g., the number 1 (10) indicates the expected value, on the –log10 scale, for the minimum (i.e. the tenth smallest) p-value).

Table S1 Minor allele frequencies of the SNPs in the region of Chromosome 4p

| No. | Chr. | SNP | Position | Minor allele | MAF | Gene |
| --- | --- | --- | --- | --- | --- | --- |
| 1 | 4 | rs7686755 | 8988690 | T | 0.311 | LOC728429 |
| 2 | 4 | rs7437149 | 9013910 | G | 0.197 | LOC728429 |
| 3 | 4 | rs6854838 | 9032348 | T | 0.076 | LOC728429 |
| 4 | 4 | rs6447935 | 9040539 | G | 0.360 | DEFB131 |
| 5 | 4 | rs11722202 | 9052992 | T | 0.467 | DEFB131 |
| 6 | 4 | rs4246697 | 9061021 | C | 0.054 | DEFB131 |
| 7 | 4 | rs9714696 | 9094398 | A | 0.090 | LOC644445 |
| 8 | 4 | rs9997518 | 9097760 | A | 0.273 | LOC644445 |
| 9 | 4 | rs9291512 | 9122563 | T | 0.358 | LOC644445 |
| 10 | 4 | rs6448742 | 9151857 | C | 0.102 | LOC728700 |
| 11 | 4 | rs10001694 | 9160192 | G | 0.259 | LOC728700 |
| 12 | 4 | rs9994111 | 9167442 | A | 0.238 | LOC728700 |
| 13 | 4 | rs6820570 | 9188118 | G | 0.148 | LOC728700 |
| 14 | 4 | rs13135278 | 9191520 | A | 0.286 | DEFB131 |
| 15 | 4 | rs10049659 | 9195862 | T | 0.148 | LOC728700 |
| 16 | 4 | rs6824806 | 9198525 | G | 0.399 | LOC728700 |
| 17 | 4 | rs12645339 | 9205713 | T | 0.041 | LOC728708 |
| 18 | 4 | rs1809401 | 9208888 | T | 0.217 | LOC728708 |
| 19 | 4 | rs7375281 | 9218604 | T | 0.168 | LOC728708 |
| 20 | 4 | rs6837106 | 9235402 | T | 0.219 | LOC728708 |
| 21 | 4 | rs10049735 | 9235507 | C | 0.135 | LOC728708 |
| 22 | 4 | rs13151183 | 9236164 | G | 0.420 | LOC728708 |
| 23 | 4 | rs13115550 | 9260511 | T | 0.047 | LOC728708 |
| 24 | 4 | rs7658414 | 9297067 | G | 0.448 | LOC728708 |
| 25 | 4 | rs4554078 | 9310642 | A | 0.161 | LOC644517 |
| 26 | 4 | rs9685887 | 9315440 | T | 0.169 | LOC644517 |
| 27 | 4 | rs13136075 | 9324066 | A | 0.376 | LOC644517 |
| 28 | 4 | rs9684176 | 9325484 | G | 0.383 | LOC644517 |
| 29 | 4 | rs11727674 | 9329079 | T | 0.436 | LOC644517 |
| 30 | 4 | rs12645989 | 9331231 | T | 0.445 | LOC644517 |
| 31 | 4 | rs12647851 | 9333617 | T | 0.074 | LOC644517 |
| 32 | 4 | rs7685513 | 9337697 | A | 0.156 | LOC644517 |
| 33 | 4 | rs10011621 | 9369337 | C | 0.310 | DRD5 |
| 34 | 4 | rs1878943 | 9375986 | A | 0.324 | DRD5 |
| 35 | 4 | rs1519097 | 9411951 | T | 0.379 | DRD5 |
| 36 | 4 | rs1533615 | 9413622 | A | 0.195 | DRD5 |
| 37 | 4 | rs16888799 | 9413654 | C | 0.149 | DRD5 |
| 38 | 4 | rs12500086 | 9418957 | G | 0.386 | SLC2A9 |
| 39 | 4 | rs1401438 | 9423554 | T | 0.118 | SLC2A9 |
| 40 | 4 | rs939132 | 9429981 | A | 0.328 | SLC2A9 |
| 41 | 4 | rs11938128 | 9433717 | A | 0.087 | SLC2A9 |
| 42 | 4 | rs1878276 | 9434043 | A | 0.402 | SLC2A9 |
| 43 | 4 | rs1519092 | 9435899 | A | 0.316 | SLC2A9 |
| 44 | 4 | rs4621429 | 9435968 | G | 0.161 | SLC2A9 |
| 45 | 4 | rs4697895 | 9437582 | C | 0.161 | SLC2A9 |
| 46 | 4 | rs1464258 | 9443205 | T | 0.423 | SLC2A9 |
| 47 | 4 | rs3775950 | 9445464 | G | 0.087 | SLC2A9 |
| 48 | 4 | rs10939552 | 9447646 | A | 0.169 | SLC2A9 |
| 49 | 4 | rs1401442 | 9448545 | G | 0.315 | SLC2A9 |
| 50 | 4 | rs950310 | 9451948 | A | 0.163 | SLC2A9 |
| 51 | 4 | rs16889496 | 9454695 | G | 0.081 | SLC2A9 |
| 52 | 4 | rs13119059 | 9456958 | C | 0.220 | SLC2A9 |
| 53 | 4 | rs11721988 | 9457997 | G | 0.163 | SLC2A9 |
| 54 | 4 | rs2176644 | 9458626 | T | 0.163 | SLC2A9 |
| 55 | 4 | rs6812007 | 9459318 | A | 0.471 | SLC2A9 |
| 56 | 4 | rs10939561 | 9460640 | G | 0.470 | SLC2A9 |
| 57 | 4 | rs4507358 | 9467054 | T | 0.392 | SLC2A9 |
| 58 | 4 | rs4575993 | 9467842 | G | 0.218 | SLC2A9 |
| 59 | 4 | rs13116446 | 9468995 | A | 0.217 | SLC2A9 |
| 60 | 4 | rs4697900 | 9469074 | G | 0.474 | SLC2A9 |
| 61 | 4 | rs1106059 | 9470610 | T | 0.220 | SLC2A9 |
| 62 | 4 | rs9684729 | 9471134 | T | 0.472 | SLC2A9 |
| 63 | 4 | rs10003673 | 9471398 | G | 0.224 | SLC2A9 |
| 64 | 4 | rs4697901 | 9472796 | G | 0.224 | SLC2A9 |
| 65 | 4 | rs4697902 | 9473034 | C | 0.253 | SLC2A9 |
| 66 | 4 | rs4697903 | 9474524 | A | 0.381 | SLC2A9 |
| 67 | 4 | rs2292917 | 9476375 | C | 0.207 | SLC2A9 |
| 68 | 4 | rs2867394 | 9481573 | A | 0.162 | SLC2A9 |
| 69 | 4 | rs13148571 | 9482953 | A | 0.209 | SLC2A9 |
| 70 | 4 | rs12647883 | 9487562 | C | 0.063 | SLC2A9 |
| 71 | 4 | rs1519098 | 9490256 | T | 0.209 | SLC2A9 |
| 72 | 4 | rs6834978 | 9490698 | C | 0.080 | SLC2A9 |
| 73 | 4 | rs6824636 | 9490949 | G | 0.297 | SLC2A9 |
| 74 | 4 | rs6847887 | 9492899 | A | 0.317 | SLC2A9 |
| 75 | 4 | rs6449090 | 9493634 | C | 0.475 | SLC2A9 |
| 76 | 4 | rs9993652 | 9497947 | C | 0.473 | SLC2A9 |
| 77 | 4 | rs4697904 | 9498092 | C | 0.234 | SLC2A9 |
| 78 | 4 | rs6818572 | 9498546 | A | 0.162 | SLC2A9 |
| 79 | 4 | rs1107710 | 9499806 | G | 0.163 | SLC2A9 |
| 80 | 4 | rs938563 | 9500096 | G | 0.382 | SLC2A9 |
| 81 | 4 | rs938562 | 9500129 | A | 0.161 | SLC2A9 |
| 82 | 4 | rs10939602 | 9501200 | C | 0.475 | SLC2A9 |
| 83 | 4 | rs4697692 | 9502295 | T | 0.162 | SLC2A9 |
| 84 | 4 | rs4697908 | 9502380 | C | 0.476 | SLC2A9 |
| 85 | 4 | rs12644047 | 9502501 | A | 0.162 | SLC2A9 |
| 86 | 4 | rs4697693 | 9504958 | C | 0.067 | SLC2A9 |
| 87 | 4 | rs4697909 | 9505858 | G | 0.487 | SLC2A9 |
| 88 | 4 | rs13136962 | 9509412 | C | 0.263 | SLC2A9 |
| 89 | 4 | rs6449100 | 9510661 | T | 0.255 | SLC2A9 |
| 90 | 4 | rs11737685 | 9512219 | G | 0.424 | SLC2A9 |
| 91 | 4 | rs10939608 | 9516434 | G | 0.498 | SLC2A9 |
| 92 | 4 | rs4389579 | 9516702 | C | 0.169 | SLC2A9 |
| 93 | 4 | rs16890728 | 9517931 | A | 0.154 | SLC2A9 |
| 94 | 4 | rs2280202 | 9518393 | C | 0.263 | SLC2A9 |
| 95 | 4 | rs2280203 | 9518657 | G | 0.489 | SLC2A9 |
| 96 | 4 | rs2280204 | 9518948 | G | 0.153 | SLC2A9 |
| 97 | 4 | rs2280205 | 9519021 | A | 0.252 | SLC2A9 |
| 98 | 4 | rs11734893 | 9519539 | A | 0.251 | SLC2A9 |
| 99 | 4 | rs13103429 | 9519733 | T | 0.251 | SLC2A9 |
| 100 | 4 | rs13108825 | 9519761 | G | 0.250 | SLC2A9 |
| 101 | 4 | rs11935405 | 9520941 | C | 0.169 | SLC2A9 |
| 102 | 4 | rs4336225 | 9520970 | T | 0.271 | SLC2A9 |
| 103 | 4 | rs11934363 | 9521199 | G | 0.169 | SLC2A9 |
| 104 | 4 | rs11722228 | 9524839 | T | 0.275 | SLC2A9 |
| 105 | 4 | rs4697695 | 9524948 | A | 0.207 | SLC2A9 |
| 106 | 4 | rs16890905 | 9525760 | C | 0.337 | SLC2A9 |
| 107 | 4 | rs10805346 | 9529445 | T | 0.300 | SLC2A9 |
| 108 | 4 | rs3733591 | 9531228 | G | 0.334 | SLC2A9 |
| 109 | 4 | rs16890979 | 9531265 | T | 0.023 | SLC2A9 |
| 110 | 4 | rs6832439 | 9533417 | A | 0.023 | SLC2A9 |
| 111 | 4 | rs2012177 | 9533975 | A | 0.485 | SLC2A9 |
| 112 | 4 | rs938553 | 9534624 | T | 0.096 | SLC2A9 |
| 113 | 4 | rs13129697 | 9536065 | G | 0.499 | SLC2A9 |
| 114 | 4 | rs881971 | 9540060 | G | 0.118 | SLC2A9 |
| 115 | 4 | rs737267 | 9543842 | T | 0.022 | SLC2A9 |
| 116 | 4 | rs4447863 | 9548067 | T | 0.407 | SLC2A9 |
| 117 | 4 | rs13139970 | 9552283 | A | 0.470 | SLC2A9 |
| 118 | 4 | rs6449144 | 9553748 | T | 0.102 | SLC2A9 |
| 119 | 4 | rs4697701 | 9555193 | A | 0.491 | SLC2A9 |
| 120 | 4 | rs2018643 | 9556219 | G | 0.117 | SLC2A9 |
| 121 | 4 | rs4339211 | 9556756 | T | 0.117 | SLC2A9 |
| 122 | 4 | rs7694997 | 9556909 | G | 0.117 | SLC2A9 |
| 123 | 4 | rs7686538 | 9557175 | T | 0.117 | SLC2A9 |
| 124 | 4 | rs4580649 | 9557559 | A | 0.117 | SLC2A9 |
| 125 | 4 | rs998676 | 9557662 | A | 0.413 | SLC2A9 |
| 126 | 4 | rs12498956 | 9559803 | A | 0.118 | SLC2A9 |
| 127 | 4 | rs4697914 | 9561364 | A | 0.118 | SLC2A9 |
| 128 | 4 | rs7378340 | 9564296 | T | 0.118 | SLC2A9 |
| 129 | 4 | rs11724510 | 9567681 | T | 0.119 | SLC2A9 |
| 130 | 4 | rs6849717 | 9567817 | T | 0.410 | SLC2A9 |
| 131 | 4 | rs6849729 | 9567830 | C | 0.119 | SLC2A9 |
| 132 | 4 | rs6843873 | 9567886 | C | 0.119 | SLC2A9 |
| 133 | 4 | rs6449157 | 9569540 | A | 0.119 | SLC2A9 |
| 134 | 4 | rs7672947 | 9570466 | C | 0.119 | SLC2A9 |
| 135 | 4 | rs17245723 | 9571316 | T | 0.119 | SLC2A9 |
| 136 | 4 | rs6839490 | 9574098 | G | 0.118 | SLC2A9 |
| 137 | 4 | rs6856127 | 9574541 | C | 0.119 | SLC2A9 |
| 138 | 4 | rs6449171 | 9575096 | C | 0.119 | SLC2A9 |
| 139 | 4 | rs6847019 | 9575347 | T | 0.096 | SLC2A9 |
| 140 | 4 | rs4505821 | 9587192 | A | 0.097 | SLC2A9 |
| 141 | 4 | rs11723970 | 9589560 | C | 0.412 | SLC2A9 |
| 142 | 4 | rs13131257 | 9590987 | T | 0.021 | SLC2A9 |
| 143 | 4 | rs13125029 | 9591127 | A | 0.118 | SLC2A9 |
| 144 | 4 | rs7660895 | 9594543 | A | 0.424 | SLC2A9 |
| 145 | 4 | rs9992406 | 9595386 | T | 0.097 | SLC2A9 |
| 146 | 4 | rs6849736 | 9595881 | A | 0.407 | SLC2A9 |
| 147 | 4 | rs3733589 | 9596422 | T | 0.406 | SLC2A9 |
| 148 | 4 | rs4502681 | 9599270 | C | 0.408 | SLC2A9 |
| 149 | 4 | rs17187075 | 9599426 | C | 0.404 | SLC2A9 |
| 150 | 4 | rs6449213 | 9603313 | C | 0.024 | SLC2A9 |
| 151 | 4 | rs3775948 | 9604280 | C | 0.428 | SLC2A9 |
| 152 | 4 | rs12499857 | 9604474 | A | 0.400 | SLC2A9 |
| 153 | 4 | rs16891923 | 9605759 | A | 0.406 | SLC2A9 |
| 154 | 4 | rs13127090 | 9606314 | A | 0.407 | SLC2A9 |
| 155 | 4 | rs10939650 | 9607538 | T | 0.500 | SLC2A9 |
| 156 | 4 | rs1014290 | 9610959 | C | 0.424 | SLC2A9 |
| 157 | 4 | rs4622999 | 9612493 | G | 0.400 | SLC2A9 |
| 158 | 4 | rs7657096 | 9613098 | G | 0.472 | SLC2A9 |
| 159 | 4 | rs10022499 | 9615635 | C | 0.492 | SLC2A9 |
| 160 | 4 | rs10016075 | 9615761 | G | 0.492 | SLC2A9 |
| 161 | 4 | rs4543113 | 9617403 | G | 0.122 | SLC2A9 |
| 162 | 4 | rs6845554 | 9622271 | T | 0.122 | SLC2A9 |
| 163 | 4 | rs6827754 | 9627251 | C | 0.122 | SLC2A9 |
| 164 | 4 | rs2240723 | 9630249 | A | 0.465 | SLC2A9 |
| 165 | 4 | rs6820230 | 9636640 | T | 0.101 | SLC2A9 |
| 166 | 4 | rs6449237 | 9636741 | G | 0.101 | SLC2A9 |
| 167 | 4 | rs10939663 | 9641614 | G | 0.078 | SLC2A9 |
| 168 | 4 | rs3733585 | 9645437 | C | 0.412 | SLC2A9 |
| 169 | 4 | rs9291645 | 9647352 | A | 0.101 | SLC2A9 |
| 170 | 4 | rs12508991 | 9650202 | T | 0.412 | SLC2A9 |
| 171 | 4 | rs4391034 | 9658798 | C | 0.102 | SLC2A9 |
| 172 | 4 | rs733175 | 9659239 | C | 0.488 | SLC2A9 |
| 173 | 4 | rs6834555 | 9671424 | G | 0.484 | WDR1 |
| 174 | 4 | rs6820756 | 9671947 | A | 0.484 | WDR1 |
| 175 | 4 | rs2241469 | 9689560 | C | 0.347 | WDR1 |
| 176 | 4 | rs10516200 | 9691254 | C | 0.463 | WDR1 |
| 177 | 4 | rs2241480 | 9698861 | C | 0.464 | WDR1 |
| 178 | 4 | rs2241482 | 9708912 | A | 0.345 | WDR1 |
| 179 | 4 | rs6830786 | 9710541 | T | 0.464 | WDR1 |
| 180 | 4 | rs717615 | 9713768 | T | 0.473 | WDR1 |
| 181 | 4 | rs4697922 | 9719703 | T | 0.341 | WDR1 |
| 182 | 4 | rs10516201 | 9733039 | C | 0.130 | WDR1 |
| 183 | 4 | rs3886038 | 9734353 | A | 0.130 | WDR1 |
| 184 | 4 | rs7699512 | 9734906 | C | 0.483 | WDR1 |
| 185 | 4 | rs7699671 | 9734972 | T | 0.184 | WDR1 |
| 186 | 4 | rs4619888 | 9736076 | C | 0.166 | WDR1 |
| 187 | 4 | rs11724184 | 9736399 | G | 0.329 | WDR1 |
| 188 | 4 | rs10001106 | 9736539 | C | 0.461 | WDR1 |
| 189 | 4 | rs11722643 | 9736582 | T | 0.329 | WDR1 |
| 190 | 4 | rs16893786 | 9737016 | T | 0.286 | WDR1 |
| 191 | 4 | rs12502368 | 9739051 | A | 0.326 | WDR1 |
| 192 | 4 | rs12501508 | 9741030 | G | 0.402 | WDR1 |
| 193 | 4 | rs12501597 | 9741235 | T | 0.128 | WDR1 |
| 194 | 4 | rs17198113 | 9747568 | T | 0.122 | WDR1 |
| 195 | 4 | rs11732828 | 9747844 | C | 0.146 | WDR1 |
| 196 | 4 | rs17198197 | 9748255 | A | 0.160 | WDR1 |
| 197 | 4 | rs10022911 | 9749649 | G | 0.023 | WDR1 |
| 198 | 4 | rs4697931 | 9754394 | T | 0.152 | WDR1 |
| 199 | 4 | rs737601 | 9762331 | A | 0.484 | WDR1 |
| 200 | 4 | rs2241465 | 9768118 | C | 0.470 | WDR1 |
| 201 | 4 | rs2241466 | 9768201 | C | 0.350 | WDR1 |
| 202 | 4 | rs4697942 | 9772125 | G | 0.350 | WDR1 |
| 203 | 4 | rs4697944 | 9774658 | G | 0.353 | WDR1 |
| 204 | 4 | rs16894270 | 9774877 | T | 0.228 | WDR1 |
| 205 | 4 | rs17385112 | 9776630 | G | 0.025 | WDR1 |
| 206 | 4 | rs1001216 | 9777947 | G | 0.215 | WDR1 |
| 207 | 4 | rs6835689 | 9781577 | T | 0.175 | WDR1 |
| 208 | 4 | rs4697952 | 9782117 | A | 0.466 | WDR1 |
| 209 | 4 | rs7657551 | 9788407 | T | 0.482 | WDR1 |
| 210 | 4 | rs6449342 | 9788867 | A | 0.482 | WDR1 |
| 211 | 4 | rs956312 | 9797424 | C | 0.479 | WDR1 |
| 212 | 4 | rs1860903 | 9805951 | T | 0.121 | WDR1 |
| 213 | 4 | rs2868420 | 9812095 | G | 0.473 | WDR1 |
| 214 | 4 | rs9990501 | 9813691 | A | 0.480 | WDR1 |
| 215 | 4 | rs4697977 | 9819751 | C | 0.474 | WDR1 |
| 216 | 4 | rs4697980 | 9849276 | T | 0.359 | WDR1 |
| 217 | 4 | rs2098230 | 9852022 | G | 0.362 | WDR1 |
| 218 | 4 | rs11732042 | 9855546 | T | 0.088 | WDR1 |
| 219 | 4 | rs1860912 | 9869575 | T | 0.363 | WDR1 |
| 220 | 4 | rs1541522 | 9872343 | T | 0.362 | WDR1 |
| 221 | 4 | rs10489076 | 9879946 | A | 0.447 | WDR1 |
| 222 | 4 | rs13134726 | 9880217 | C | 0.348 | WDR1 |
| 223 | 4 | rs10489075 | 9881191 | T | 0.349 | WDR1 |
| 224 | 4 | rs10489074 | 9881703 | T | 0.147 | WDR1 |
| 225 | 4 | rs10489073 | 9881886 | T | 0.147 | WDR1 |
| 226 | 4 | rs10489072 | 9882342 | G | 0.499 | WDR1 |
| 227 | 4 | rs10939814 | 9882427 | T | 0.441 | WDR1 |
| 228 | 4 | rs1860910 | 9884568 | C | 0.437 | WDR1 |
| 229 | 4 | rs10805364 | 9884616 | G | 0.119 | WDR1 |
| 230 | 4 | rs6833142 | 9885080 | G | 0.494 | WDR1 |
| 231 | 4 | rs17473279 | 9885348 | G | 0.013 | WDR1 |
| 232 | 4 | rs734662 | 9890239 | T | 0.363 | ZNF518B |
| 233 | 4 | rs4698013 | 9894479 | T | 0.363 | ZNF518B |
| 234 | 4 | rs4698014 | 9895399 | T | 0.121 | ZNF518B |
| 235 | 4 | rs6449438 | 9900161 | T | 0.404 | ZNF518B |
| 236 | 4 | rs12650204 | 9913539 | T | 0.254 | ZNF518B |
| 237 | 4 | rs1468692 | 9914873 | C | 0.405 | ZNF518B |
| 238 | 4 | rs6449450 | 9920985 | A | 0.357 | ZNF518B |
| 239 | 4 | rs714086 | 9922140 | A | 0.132 | ZNF518B |
| 240 | 4 | rs727995 | 9923275 | C | 0.493 | ZNF518B |
| 241 | 4 | rs714436 | 9923765 | G | 0.025 | ZNF518B |
| 242 | 4 | rs7666514 | 9925977 | A | 0.058 | ZNF518B |
| 243 | 4 | rs2024277 | 9927450 | G | 0.228 | ZNF518B |
| 244 | 4 | rs12506625 | 9931283 | G | 0.421 | ZNF518B |
| 245 | 4 | rs993173 | 9933033 | G | 0.270 | ZNF518B |
| 246 | 4 | rs9291683 | 9933258 | A | 0.297 | ZNF518B |
| 247 | 4 | rs1558488 | 9936512 | C | 0.316 | ZNF518B |
| 248 | 4 | rs4698036 | 9940392 | G | 0.133 | ZNF518B |
| 249 | 4 | rs2007103 | 9950353 | T | 0.476 | ZNF518B |
| 250 | 4 | rs17420080 | 9954646 | T | 0.025 | ZNF518B |
| 251 | 4 | rs2192084 | 9956149 | A | 0.477 | ZNF518B |
| 252 | 4 | rs2192083 | 9956862 | A | 0.351 | ZNF518B |
| 253 | 4 | rs2098235 | 9963167 | T | 0.419 | ZNF518B |
| 254 | 4 | rs4235361 | 9974191 | A | 0.423 | ZNF518B |
| 255 | 4 | rs6851536 | 9982064 | C | 0.484 | ZNF518B |
| 256 | 4 | rs10938772 | 9984426 | G | 0.115 | ZNF518B |
| 257 | 4 | rs4698043 | 9987700 | G | 0.482 | ZNF518B |
| 258 | 4 | rs7677806 | 9992103 | C | 0.483 | ZNF518B |
| 259 | 4 | rs4302456 | 9995772 | C | 0.111 | ZNF518B |
| 260 | 4 | rs9990427 | 9997411 | A | 0.362 | ZNF518B |
| 261 | 4 | rs9990701 | 9997708 | A | 0.482 | ZNF518B |
| 262 | 4 | rs11724536 | 9999387 | T | 0.359 | ZNF518B |
| 263 | 4 | rs4698048 | 10007733 | G | 0.405 | ZNF518B |
| 264 | 4 | rs4607209 | 10009254 | G | 0.418 | ZNF518B |
| 265 | 4 | rs4530622 | 10011936 | C | 0.338 | ZNF518B |
| 266 | 4 | rs7677318 | 10015389 | G | 0.354 | ZNF518B |
| 267 | 4 | rs4643800 | 10016670 | T | 0.024 | ZNF518B |
| 268 | 4 | rs11728025 | 10017319 | G | 0.025 | ZNF518B |
| 269 | 4 | rs7656072 | 10018939 | G | 0.497 | ZNF518B |
| 270 | 4 | rs4698050 | 10019846 | T | 0.025 | ZNF518B |
| 271 | 4 | rs7661365 | 10023006 | A | 0.352 | ZNF518B |
| 272 | 4 | rs7683253 | 10028563 | T | 0.189 | ZNF518B |
| 273 | 4 | rs4552452 | 10033421 | G | 0.322 | ZNF518B |
| 274 | 4 | rs4273464 | 10044442 | A | 0.257 | ZNF518B |
| 275 | 4 | rs10003864 | 10046489 | C | 0.250 | ZNF518B |
| 276 | 4 | rs10938799 | 10052523 | A | 0.180 | ZNF518B |
| 277 | 4 | rs10016702 | 10056738 | G | 0.136 | ZNF518B |
| 278 | 4 | rs4333176 | 10084378 | A | 0.403 | ZNF518B |
| 279 | 4 | rs6835433 | 10094085 | C | 0.323 | MIST |
| 280 | 4 | rs11737601 | 10097666 | G | 0.202 | MIST |
| 281 | 4 | rs11722849 | 10097824 | C | 0.246 | MIST |
| 282 | 4 | rs10488945 | 10099376 | T | 0.410 | MIST |
| 283 | 4 | rs3749558 | 10103101 | T | 0.187 | MIST |
| 284 | 4 | rs6819820 | 10107928 | A | 0.392 | MIST |
| 285 | 4 | rs17467273 | 10109529 | C | 0.182 | MIST |
| 286 | 4 | rs5000857 | 10111432 | G | 0.491 | MIST |
| 287 | 4 | rs10488947 | 10112850 | G | 0.210 | MIST |
| 288 | 4 | rs17467315 | 10115331 | C | 0.271 | MIST |
| 289 | 4 | rs13141385 | 10117502 | A | 0.183 | MIST |
| 290 | 4 | rs13109005 | 10119979 | G | 0.397 | MIST |
| 291 | 4 | rs1004327 | 10120581 | C | 0.319 | MIST |
| 292 | 4 | rs1004329 | 10121094 | G | 0.399 | MIST |
| 293 | 4 | rs13125670 | 10122170 | T | 0.240 | MIST |
| 294 | 4 | rs13125086 | 10129791 | C | 0.133 | MIST |
| 295 | 4 | rs977125 | 10131357 | C | 0.047 | MIST |
| 296 | 4 | rs2041216 | 10132188 | C | 0.384 | MIST |
| 297 | 4 | rs12641877 | 10137324 | G | 0.382 | MIST |
| 298 | 4 | rs7657102 | 10138618 | G | 0.141 | MIST |
| 299 | 4 | rs10488948 | 10145642 | A | 0.313 | MIST |
| 300 | 4 | rs12508896 | 10154951 | G | 0.484 | MIST |
| 301 | 4 | rs11939512 | 10155457 | C | 0.137 | MIST |
| 302 | 4 | rs13112750 | 10156441 | C | 0.442 | MIST |
| 303 | 4 | rs6848730 | 10160658 | G | 0.230 | MIST |
| 304 | 4 | rs1974584 | 10166959 | T | 0.423 | MIST |
| 305 | 4 | rs4698069 | 10168139 | G | 0.408 | MIST |
| 306 | 4 | rs7655244 | 10177311 | T | 0.058 | MIST |
| 307 | 4 | rs17467553 | 10181965 | T | 0.057 | MIST |
| 308 | 4 | rs12643937 | 10187078 | T | 0.161 | MIST |
| 309 | 4 | rs2014303 | 10194954 | A | 0.075 | MIST |
| 310 | 4 | rs11934521 | 10198651 | A | 0.076 | MIST |
| 311 | 4 | rs4697759 | 10201089 | T | 0.139 | MIST |
| 312 | 4 | rs2531178 | 10210232 | G | 0.434 | MIST |
| 313 | 4 | rs4697760 | 10211016 | T | 0.180 | MIST |
| 314 | 4 | rs2720354 | 10222550 | A | 0.225 | MIST |
| 315 | 4 | rs2720372 | 10226723 | C | 0.227 | MIST |
| 316 | 4 | rs978591 | 10227767 | C | 0.227 | MIST |
| 317 | 4 | rs2531198 | 10229443 | T | 0.225 | MIST |
| 318 | 4 | rs2531208 | 10241502 | T | 0.224 | MIST |
| 319 | 4 | rs4128567 | 10245939 | T | 0.227 | MIST |
| 320 | 4 | rs6836007 | 10256682 | A | 0.294 | MIST |
| 321 | 4 | rs10516207 | 10259791 | T | 0.110 | MIST |
| 322 | 4 | rs13107947 | 10260715 | T | 0.464 | MIST |
| 323 | 4 | rs12233843 | 10264316 | A | 0.485 | MIST |
| 324 | 4 | rs13119277 | 10267227 | T | 0.463 | MIST |
| 325 | 4 | rs10516208 | 10270832 | A | 0.480 | MIST |
| 326 | 4 | rs6855114 | 10271488 | T | 0.286 | MIST |
| 327 | 4 | rs6448177 | 10275370 | C | 0.484 | MIST |
| 328 | 4 | rs3935222 | 10275732 | A | 0.500 | MIST |
| 329 | 4 | rs12502030 | 10276574 | T | 0.186 | MIST |
| 330 | 4 | rs4323097 | 10277889 | A | 0.186 | MIST |
| 331 | 4 | rs13136927 | 10278620 | C | 0.371 | MIST |
| 332 | 4 | rs13115026 | 10279144 | A | 0.313 | MIST |
| 333 | 4 | rs4697777 | 10284128 | C | 0.494 | MIST |
| 334 | 4 | rs10033332 | 10289023 | A | 0.447 | MIST |
| 335 | 4 | rs6827843 | 10297735 | A | 0.373 | MIST |
| 336 | 4 | rs12510590 | 10299141 | T | 0.379 | MIST |
| 337 | 4 | rs6448182 | 10302320 | C | 0.343 | MIST |
| 338 | 4 | rs11725929 | 10304089 | T | 0.272 | MIST |
| 339 | 4 | rs4697648 | 10307363 | A | 0.198 | MIST |
| 340 | 4 | rs6819946 | 10311254 | T | 0.403 | MIST |
| 341 | 4 | rs7658545 | 10311487 | G | 0.087 | MIST |
| 342 | 4 | rs4549371 | 10316606 | C | 0.117 | MIST |
| 343 | 4 | rs6831973 | 10318464 | T | 0.402 | MIST |
| 344 | 4 | rs6844834 | 10320027 | T | 0.205 | MIST |
| 345 | 4 | rs4561924 | 10323555 | C | 0.203 | MIST |
| 346 | 4 | rs4697651 | 10330531 | T | 0.139 | MIST |
| 347 | 4 | rs16872571 | 10335951 | T | 0.447 | MIST |
| 348 | 4 | rs11736462 | 10337494 | G | 0.388 | MIST |
| 349 | 4 | rs4697653 | 10337857 | G | 0.301 | MIST |
| 350 | 4 | rs4334748 | 10338484 | T | 0.300 | MIST |
| 351 | 4 | rs12644305 | 10349365 | C | 0.090 | MIST |
| 352 | 4 | rs12650576 | 10353646 | T | 0.088 | MIST |
| 353 | 4 | rs12640839 | 10354029 | T | 0.088 | MIST |
| 354 | 4 | rs10016050 | 10356671 | C | 0.181 | MIST |
| 355 | 4 | rs16872837 | 10357479 | G | 0.181 | MIST |
| 356 | 4 | rs10007887 | 10358169 | T | 0.268 | MIST |
| 357 | 4 | rs4420973 | 10360841 | C | 0.340 | MIST |
| 358 | 4 | rs4073342 | 10383692 | C | 0.339 | MIST |
| 359 | 4 | rs16873628 | 10445143 | C | 0.311 | MIST |
| 360 | 4 | rs12645385 | 10449748 | T | 0.084 | MIST |
| 361 | 4 | rs4257647 | 10454305 | G | 0.130 | MIST |
| 362 | 4 | rs7673890 | 10456401 | A | 0.268 | MIST |
| 363 | 4 | rs10516213 | 10480753 | C | 0.051 | MIST |
| 364 | 4 | rs959190 | 10481839 | G | 0.063 | MIST |
| 365 | 4 | rs1483656 | 10485756 | T | 0.053 | MIST |
| 366 | 4 | rs7698615 | 10486321 | G | 0.212 | MIST |
| 367 | 4 | rs10516215 | 10487213 | C | 0.263 | MIST |
| 368 | 4 | rs2170752 | 10493178 | C | 0.486 | MIST |
| 369 | 4 | rs16874378 | 10495622 | G | 0.167 | MIST |
| 370 | 4 | rs11945933 | 10497503 | G | 0.212 | MIST |
| 371 | 4 | rs4697659 | 10498345 | C | 0.264 | MIST |
| 372 | 4 | rs1483664 | 10502351 | G | 0.082 | MIST |
| 373 | 4 | rs959233 | 10511257 | G | 0.419 | MIST |
| 374 | 4 | rs7660096 | 10512103 | G | 0.138 | MIST |
| 375 | 4 | rs2869764 | 10514288 | G | 0.138 | MIST |
| 376 | 4 | rs10011464 | 10515465 | T | 0.417 | MIST |
| 377 | 4 | rs10028469 | 10518323 | T | 0.136 | MIST |
| 378 | 4 | rs6847172 | 10534156 | G | 0.299 | MIST |
| 379 | 4 | rs4697808 | 10540803 | G | 0.048 | MIST |
| 380 | 4 | rs12643516 | 10548137 | T | 0.416 | MIST |
| 381 | 4 | rs6837093 | 10552101 | G | 0.416 | MIST |
| 382 | 4 | rs17281029 | 10552595 | A | 0.123 | MIST |
| 383 | 4 | rs13124128 | 10558923 | T | 0.267 | MIST |
| 384 | 4 | rs12510358 | 10560191 | T | 0.417 | MIST |
| 385 | 4 | rs1466529 | 10561639 | T | 0.463 | MIST |
| 386 | 4 | rs6821267 | 10562303 | G | 0.318 | MIST |
| 387 | 4 | rs1564737 | 10563592 | C | 0.407 | MIST |
| 388 | 4 | rs2869770 | 10575992 | C | 0.421 | MIST |
| 389 | 4 | rs6448400 | 10577619 | A | 0.434 | MIST |
| 390 | 4 | rs9291474 | 10579909 | C | 0.475 | MIST |
| 391 | 4 | rs10009111 | 10580521 | A | 0.052 | MIST |
| 392 | 4 | rs10011697 | 10580930 | A | 0.052 | MIST |
| 393 | 4 | rs10516217 | 10582359 | A | 0.038 | MIST |
| 394 | 4 | rs10516218 | 10584514 | A | 0.179 | MIST |
| 395 | 4 | rs2128866 | 10584994 | C | 0.218 | MIST |
| 396 | 4 | rs2046359 | 10598106 | T | 0.370 | MIST |
| 397 | 4 | rs4697823 | 10604526 | A | 0.448 | MIST |
| 398 | 4 | rs2128863 | 10611626 | C | 0.260 | MIST |
| 399 | 4 | rs875394 | 10620696 | T | 0.014 | MIST |
| 400 | 4 | rs9291483 | 10625831 | C | 0.295 | MIST |
| 401 | 4 | rs6448447 | 10628445 | T | 0.164 | MIST |
| 402 | 4 | rs4373155 | 10631897 | G | 0.216 | MIST |
| 403 | 4 | rs4622992 | 10636912 | T | 0.193 | MIST |

(Note: In this region, nineteen SNPs with MAF < 0.01 were removed.)


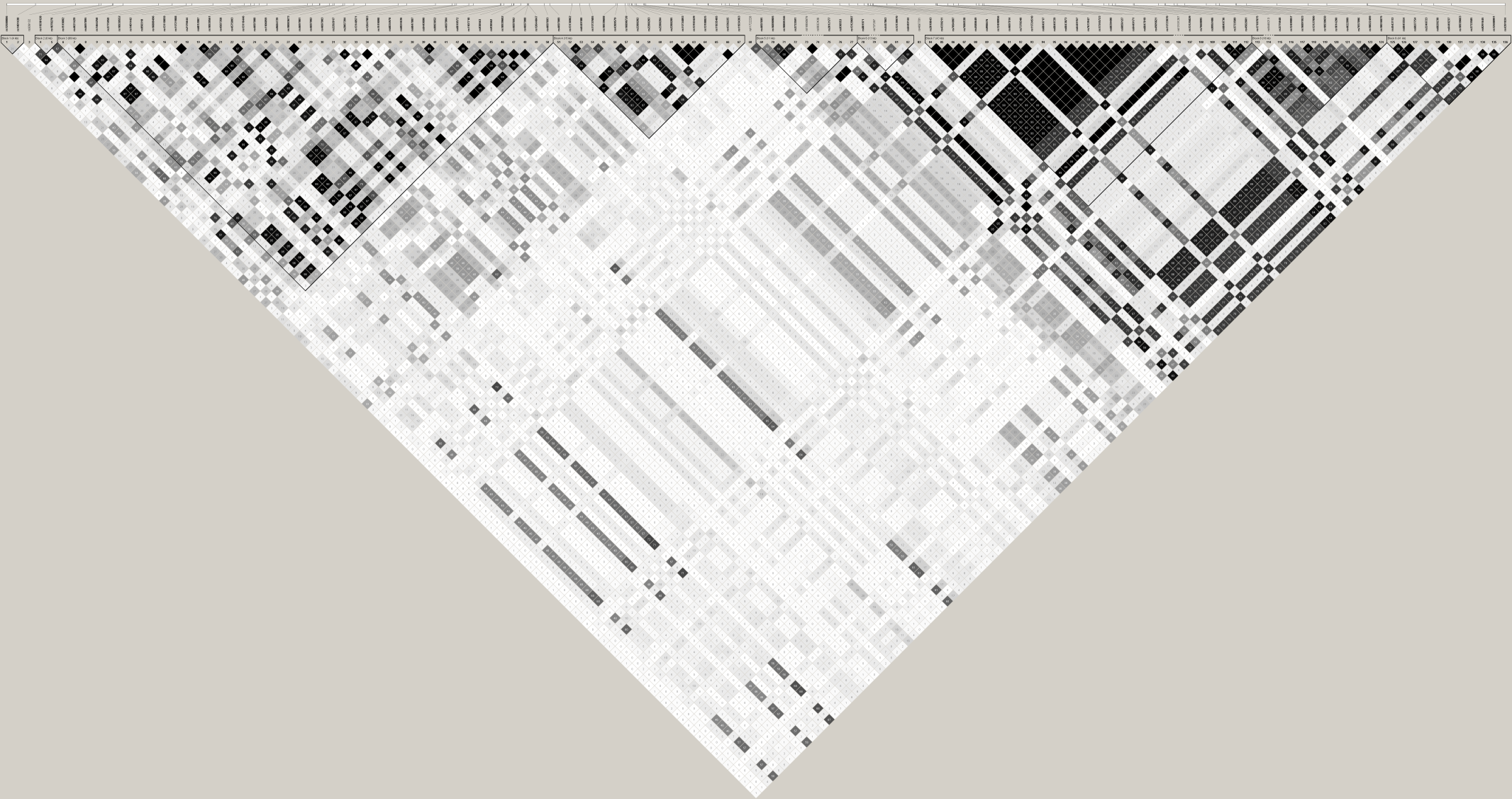


Figure S3.1 LD plots for *SLC2A9* among 259 Asian CP trios. Black squares represent r^2^=1; gray squares represent 0<r^2^<1; white squares represent r^2^=0.


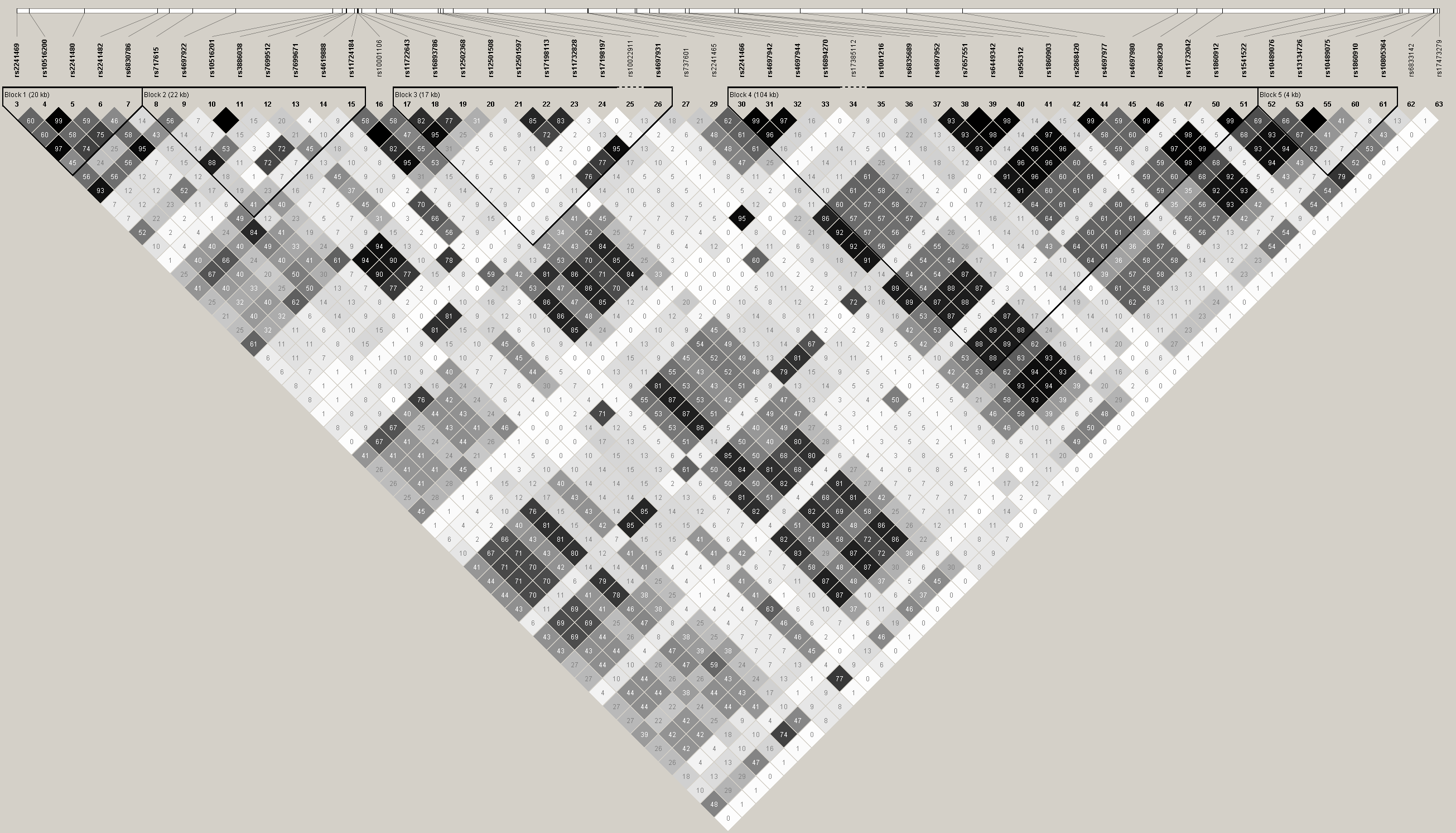


Figure S3.2 LD plots for *WDR1* among 259 Asian CP trios. Black squares represent r^2^=1; gray squares represent 0<r^2^<1; white squares represent r^2^=0.


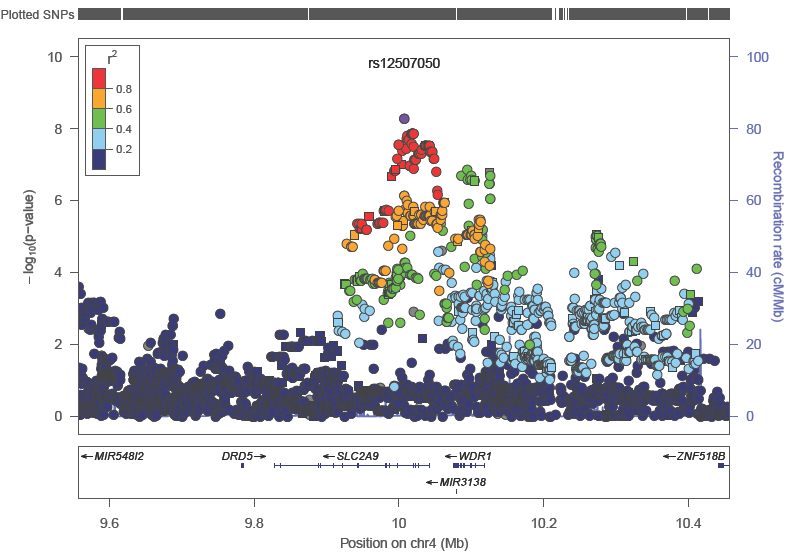


Figure S4: *P* values from likelihood ratio test with 1 degree of freedom testing for GxETS interaction after including the imputed SNPs among Asian CP trios. Circles represent imputed genotypes using 1000 Genomes as a reference population and squares represent observed SNPs.
